# Clues for improvement of research in objective structured clinical examination

**DOI:** 10.1080/10872981.2024.2370617

**Published:** 2024-06-27

**Authors:** Jean Philippe Foy, Laure Serresse, Maxens Decavèle, Manon Allaire, Nadia Nathan, Marie Christine Renaud, Nada Sabourdin, Yasmine Souala-Chalet, Yanis Tamzali, Jessica Taytard, Mélanie Tran, Fleur Cohen, Hugo Bottemanne, Antoine Monsel

**Affiliations:** a OSCE Research Group, Sorbonne Université, Paris, France; b Department of Maxillo-facial Surgery, Hôpital Pitié-Salpêtrière, Assistance Publique des Hôpitaux de Paris, Sorbonne Université, GRC 33 (RIC), INSERM UMRS 938, Centre de Recherche de Saint Antoine, Team Cancer Biology and Therapeutics, Paris, France; c INSERM, UMRS1158 Neurophysiologie Respiratoire Expérimentale et Clinique; Service hexa-site Equipe Soins Palliatif, Accompagnement et Soins de Support, Hôpitaux Charles-Foix, Pitié-Salpêtrière, Rothschild, Tenon, Trousseau/La Roche-Guyon, Groupe Hospitalier Universitaire APHP Sorbonne Université, Sorbonne Université, Paris, France; d INSERM, UMRS1158 Neurophysiologie Respiratoire Expérimentale et Clinique, Sorbonne Université, Paris, France; e Service de Médecine Intensive et Réanimation (Département R3S), Groupe Hospitalier Universitaire APHP-Sorbonne Université, site Pitié-Salpêtrière, Paris, France; f Department of Hepato-gastroenterology, Hôpital Pitié-Salpêtrière, Assistance Publique des Hôpitaux de Paris, Paris, France; g Team Proliferation, Stress and Liver Physiopathology, INSERM UMRS 1138, Centre de Recherche des Cordeliers, Paris, France; h Pediatric Pulmonology Department and Reference Center for Rare Lung Diseases RespiRare, Armand Trousseau Hospital, Paris, France; i Laboratory of Childhood Genetic Diseases, Armand Trousseau Hospital, Paris, France & Sorbonne Université, Assistance Publique – Hôpitaux de Paris, Sorbonne Université, Inserm UMR_S933, Paris, France; j Department of Anesthesiology and Intensive Care, CHU Armand Trousseau, GRC 29, Sorbonne University, APHP, University of Paris: Pharmacologie et Evaluation des Thérapeutiques chez L’enfant et la Femme Enceinte, Paris, France; k Department of Gynaecology and Obstetrics, Pitié-Salpêtrière Hospital, DMU Origyne, Assistance Publique - Hôpitaux de Paris (AP-HP), Sorbonne Université, Paris, France; l Medical and Surgical Department of Kidney Transplantation, Department of Infectious and Tropical Diseases, Pitié-Salpêtrière Hospital, DMU Origyne, Assistance Publique - Hôpitaux de Paris (AP-HP), Sorbonne Université, Paris, France; m Pediatric Pulmonology Department and Reference Center for Rare Lung Diseases RespiRare, Armand Trousseau Hospital, Assistance Publique – Hôpitaux de Paris, Paris, France; n Enruophysiologie Respiratoire expérimentale et clinique, Sorbonne Université, INSERM UMR_S1158, Paris, France; o Department of Internal Medicine, Institut E3M, CIMI-Paris, Faculty of Medicine, National Reference Centre of Systemic Lupus, Antiphospholipid Syndrome, and Other Autoimmune Diseases, Sorbonne University, AP-HP, Pitié Salpêtrière, Boulevard de l’Hôpital, Paris, France; p Department of Psychiatry, Pitie-Salpetrière Hospital, DMU Neuroscience, Assistance Publique – Hôpitaux de Paris, Sorbonne Université, Institut du Cerveau – Paris Brain Institute, UMR7225/UMR8011, CNRS/INSERM, Paris, France; q Multidisciplinary Intensive Care Unit, Department of Anesthesiology and Critical Care, La Pitié-Salpêtrière Hospital, Assistance-Publique Hôpitaux de Paris, Sorbonne University of Paris, Paris, France; r Unité mixte de recherche (UMR)-S 959, Immunology-Immunopathology-Immunotherapy (I3), Institut National de La Santé et de La Recherche Médicale (INSERM), Paris, France; s Biotherapy (CIC-BTi) and Inflammation-Immunopathology-Biotherapy Department (DHU i2B), Hôpital Pitié-Salpêtrière, Assistance Publique-Hôpitaux de Paris, Paris, France

**Keywords:** OSCE, objective structured clinical examination, scientometry, bibliometry, scientific network

## Abstract

While objective clinical structured examination (OSCE) is a worldwide recognized and effective method to assess clinical skills of undergraduate medical students, the latest Ottawa conference on the assessment of competences raised vigorous debates regarding the future and innovations of OSCE. This study aimed to provide a comprehensive view of the global research activity on OSCE over the past decades and to identify clues for its improvement. We performed a bibliometric and scientometric analysis of OSCE papers published until March 2024. We included a description of the overall scientific productivity, as well as an unsupervised analysis of the main topics and the international scientific collaborations. A total of 3,224 items were identified from the Scopus database. There was a sudden spike in publications, especially related to virtual/remote OSCE, from 2020 to 2024. We identified leading journals and countries in terms of number of publications and citations. A co-occurrence term network identified three main clusters corresponding to different topics of research in OSCE. Two connected clusters related to OSCE performance and reliability, and a third cluster on student’s experience, mental health (anxiety), and perception with few connections to the two previous clusters. Finally, the United States, the United Kingdom, and Canada were identified as leading countries in terms of scientific publications and collaborations in an international scientific network involving other European countries (the Netherlands, Belgium, Italy) as well as Saudi Arabia and Australia, and revealed the lack of important collaboration with Asian countries. Various avenues for improving OSCE research have been identified: i) developing remote OSCE with comparative studies between live and remote OSCE and issuing international recommendations for sharing remote OSCE between universities and countries; ii) fostering international collaborative studies with the support of key collaborating countries; iii) investigating the relationships between student performance and anxiety.

## Introduction

Objective structured clinical examination (OSCE), first described in 1975 [1], is an examination method dedicated to assess clinical competence in under- or post-graduate medical and allied healthcare students through a series of simulated tasks and interactions [2]. Since its initial description, OSCE method has been the object of worldwide research, with leading countries in North America and the United Kingdom. Many previously published data exist regarding the reliability and validity of OSCE to discriminate students [3,4] as well as the determinants of students’ performances during OSCE [5,6]. However, only scarce studies explored student’s perception during OSCE, especially the relationship between anxiety and students’ performances during OSCE [7] despite the well-known effects of anxiety on cognitive functioning [8,9].

Furthermore, besides the exponential increase in OSCE scientific production during the past decade, the COVID-19 pandemic led to innovations such as remote OSCE and suggested the development of international collaborations. More recently, OSCE was at the heart of a debate during the latest Ottawa Conference in 2022 on the Assessment of Competence in Medicine and the Healthcare Professions [10]. Issues such as abandoning high-stakes OSCE and replacing them with some forms of workplace-based assessment (WBA) were widely discussed. These debates highlighted the need for additional research and improvement, especially regarding OSCE-related innovations such as virtual OSCE.

As previously described in many scientific disciplines [11–13], bibliometric and scientometric analysis is relevant to inventory published articles and provide an analytic overview on research production in a specific field. Assessing the quantity and quality of scientific publications in OSCE may help to identify research gaps and pave the way toward future programs [14–16]. To date, despite accumulating scientific production, a scientometric analysis of OSCE research production is still lacking. Guidelines for informative and publication-worthy scientometric analysis of literature reporting have been published [17]. According to these guidelines, research in OSCE seems to fulfil appropriateness criteria for scientometric analysis since OSCE 1) corresponds to a mature research field with a first publication in 1979 with increasing publications, 2) covers an appropriate number of publications (i.e., more than 1,000 but not too excessive), and 3) is an unambiguous ‘medical subject heading’ for literature search.

Thus, we proposed to perform a bibliometric and scientometric analysis of research on OSCE worldwide. Our specific goal was to assess the assumption that research in OSCE is experiencing a paradigm shift towards 1) the development of remote/virtual OSCE; 2) the need for more considering student’s perception; 3) the need to develop the international scientific network conducted by leading countries such as the United States of America, the United Kingdom, and Canada.

## Material and methods

### Database and search strategy

We performed a literature search using Elsevier’s Scopus database for OSCE. The Scopus database was selected because it offers more coverage for citation analysis and covers a wider journal range, especially related to human social sciences, and more scientific publications compared to other databases [18–21]. Moreover, Scopus has the advantage of providing advanced export functionality of structured data. Using the document ‘search’ functionality, we performed a search query for all publications containing the following words in their titles, abstracts, and/or keywords: ‘objective structured clinical examination’. The acronym ‘OSCE’ was not included in the literature search as it also refers to the Organization for Security and Co-operation in Europe. The research was limited to all published or in-press documents until March 10^th^ 2024. Using Scopus ‘export’ functionalities, we exported the number of publications per journal, as well as the type of publication and the journal. Cleaning and filtering data was performed using the OpenRefine Software with the General Refine Expression Language (GREL), in order to detect duplicates and misspelled elements.

### Bibliometric performance indicators

To assess each journal’s productivity, we analyzed the number of items per journal. As previously described [12,13], we also defined the publishing rate (pR) as the number of items divided by publication time in years (i.e., number of years from the first to the last year of publication until 2024). Finally, we defined the citation rate (cR) of each journal and each country as the sum of the total times cited divided by the total number of items published by the journal or country.

### Network map of the literature on OSCE

The VOSviewer software [22] (versions 1.6.18) was used to construct and visualize a co-occurrence network of relevant terms related to OSCE research. We performed a term co-occurrence map based on text data extracted from the abstract field of Scopus, using the full counting method (i.e., all occurrences of a term in a document are counted). The most relevant terms were selected using a minimum number of occurrences of 40. As described in similar studies [23–25], this threshold was arbitrarily chosen to obtain a significant and reasonable number of ‘relevant terms’ (~300). Limiting the number of terms allows constructing a more readable map of co-occurrent terms. A relevance score was computed for each term, and the 60% most relevant terms (default parameter) were used for mapping. Mapping was automatically done using the VOS clustering technique and visualized using the ‘network visualization’ functionality.

### International scientific network

The bibliometric dataset exported from Scopus was cleaned using the OpenRefine opensource software to retrieve the country corresponding to each publication. More precisely, the General Refine Expression Language (GREL) was used to extract the country name corresponding to each author’s affiliation for one publication.

We performed a Density Equalizing Map Projection (DEMP) to visualize the number of publications on OSCE by country. The DEMP was illustrated using ScapeToad [26], and then QGIS software [27,28] was used for the addition of the color legend. Using the Gastner/Newman diffusion-based algorithm [29], map surfaces were adapted to the number of published items by country, without altering their topological relationships.

Cleaned and filtered bibliometric data, including publications per country, were then imported to Table 2 Net [30], in order to build worldwide scientific networks including all countries contributing to research in OSCE. This network was visualized using the Gephi software [31], with which we ran the ‘Map of countries’ and ‘Geolayout’ algorithms. The Gephi software was also used to calculate occurrence counts as well as ‘betweenness centrality’, a measure of how often a node lies on the shortest path between nodes in the network. Betweenness centrality is an indirect way to assess the influence a country has over the international research network.

## Results

### Bibliometric analysis

From 1979 (year of the first publication by Harden) to 2024, a total of 3,224 items were retrieved from the Scopus database, including 2819 (87.4%) articles, 182 (5.6%) reviews, 80 (2.5%) conference papers or reviews; 61 (1.9%) letters or editorials, 27 (0.8%) notes, 39 (1.2%) books or book chapters, 10 (0.3%) short surveys, 4 (0.1%) errata, one data paper, and one preprint. Most documents were published in four leading journals in the field, with more than 100 publications per journal: Medical Education, BMC Medical Education, Medical Teacher, and Academic Medicine (Table 1).

**Table 1.** Top 10 journals contributing to research on OSCE.

SOURCE TITLE	Nb of published items	Nb of citations	Publication start year	pR	cR
Medical Education	182	9770	1976	3.8	53.7
BMC Medical Education	148	2234	2001	6.4	15.1
Medical Teacher	138	3250	2004	6.9	23.6
Academic Medicine	102	3983	1989	2.9	39.0
American Journal Of Pharmaceutical Education	54	1043	1937	0.6	19.3
Teaching And Learning In Medicine	52	1635	2019	10.4	31.4
Nurse Education Today	49	1430	1981	1.1	29.2
Journal Of Surgical Education	45	548	2007	2.6	12.2
Currents In Pharmacy Teaching And Learning	40	229	2009	2.7	5.7
Journal Of Dental Education	40	597	1936	0.5	14.9

The number of citations and the citation rate (cR) are shown. Abbreviations: Nb: number; cR: citation rate.

**Figure 1.**
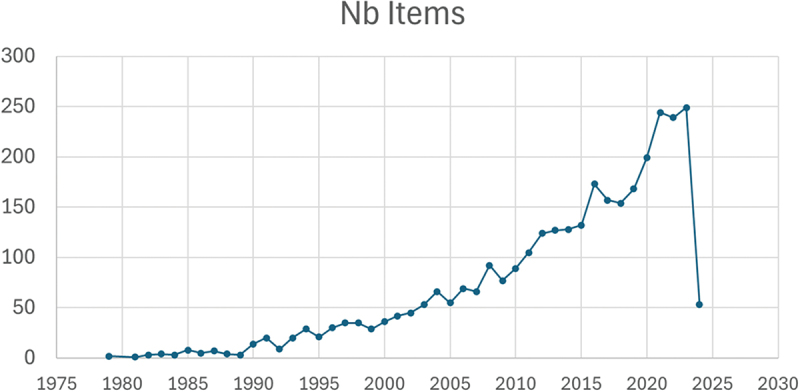
Time trend analysis of publications.

A substantial increase in publications was observed in the early 2000s. There was also a spike in OSCE-related publications from 2019 to 2022 (Figure 1), corresponding to the start of the COVID-19 pandemic. We observed that 7 of the top 10 most cited articles during the period 2020–2022 were related to remote/virtual OSCE, and one article was related to the impact of COVID-19 on evaluation of medical students, including OSCE. Conversely, before 2019, none of the top 10 most cited articles were related to remote/virtual OSCE (Table 2).

**Table 2.** Top 10 most cited studies from a literature search for OSCE using the SCOPUS database before (A) and after (B) COVID-19 pandemic.

Authors		Title	Year	Type	Source title	Nb Cit.	Nb Cit. per year
A. Period 1975–2019							
HARDEN R.M		Assessment of clinical competence using an objective structured clinical examination (OSCE)	1979	Article	Medical Education	989	22.0
Wass V		Assessment of clinical competence	2001	Review	Lancet	733	31.9
Barrows H.S.		An overview of the uses of standardized patients for teaching and evaluating clinical skills	1993	Article	Academic Medicine	658	21.2
van der Vleuten C.P.M.		Assessment of Clinical Skills With Standardized Patients: State of the Art	1990	Review	Teaching and Learning in Medicine	540	15.9
Eva K.W.		An admissions OSCE: The multiple mini-interview	2004	Article	Medical Education	479	24.0
Downing S.M.		Reliability: On the reproducibility of assessment data	2004	Review	Medical Education	476	23.8
Yedidia M.J		Effect of Communications Training on Medical Student Performance	2003	Article	JAMA	362	17.2
Newble D.		Techniques for measuring clinical competence: Objective structured clinical examinations	2004	Article	Medical Education	311	15.6
Williams R.G		Cognitive, Social and Environmental Sources of Bias in Clinical Performance Ratings	2003	Review	Teaching and Learning in Medicine	305	14.5
Alinier G		Effectiveness of intermediate-fidelity simulation training technology in undergraduate nursing education	2006	Article	Journal of Advanced Nursing	296	16.4
B. Period 2019–2024							
Choi B.	The impact of the COVID-19 pandemic on final year medical students in the United Kingdom: A national survey		2020	Article	BMC Medical Education	262	65.5
Kourgiantakis T.	Simulation in Social Work Education: A Scoping Review		2020	Article	Research on Social Work Practice	85	21.25
Lara S.	Remote Assessment of Clinical Skills During COVID-19: A Virtual, High-Stakes, Summative Pediatric Objective Structured Clinical Examination		2020	Note	Academic Pediatrics	62	15.5
Lawrence K.	Building Telemedicine Capacity for Trainees During the Novel Coronavirus Outbreak: a Case Study and Lessons Learned		2020	Article	Journal of General Internal Medicine	47	11.75
Hopwood J.	Twelve tips for conducting a virtual OSCE		2021	Article	Medical Teacher	43	14.33
Hannon P.	An objective structured clinical examination: From examination room to Zoom breakout room		2020	Article	Medical education	35	8.75
Roman P.	The escape room as evaluation method: A qualitative study of nursing students’ experiences		2020	Article	Medical Teacher	31	7.75
Li S.W.	ChatGPT outscored human candidates in a virtual objective structured clinical examination in obstetrics and gynecology		2023	Article	American Journal of Obstetrics and Gynecology	30	30
Donn J.	A pilot of a Virtual Objective Structured Clinical Examination in dental education. A response to COVID-19		2021	Article	European Journal of Dental Education	30	10
Hannan T.A.	Designing and running an online Objective Structured Clinical Examination (OSCE) on Zoom: A peer-led example		2021	Article	Medical Teacher	29	9.667

The number of citations and the citation rate (cR) are shown. Abbreviations: Nb: number; cR: citation rate.

### Term co-occurrence network

**Figure 2.**
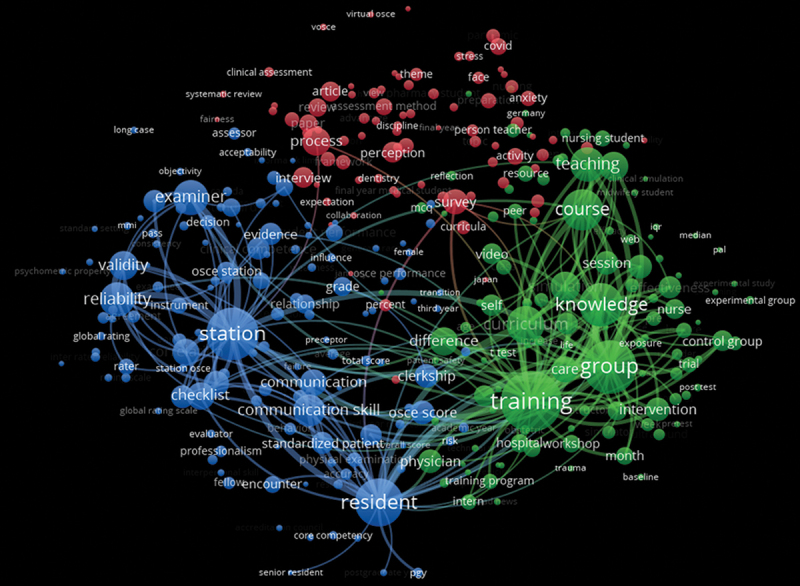
Co-occurrence network of most relevant terms related to OSCE research.

From the 51,178 terms extracted from the abstract field of the Scopus dataset, 321 relevant terms were selected. Three distinct clusters (Figure 2) were identified in this term co-occurrence network and analyzed according to the top 15 most occurrent terms. Cluster 1 (blue) included terms related to relevance and robustness of OSCE: station, reliability, communication, skill, validity, checklist, OSCE score, clerkship, rating, evidence, and standardized patient. Cluster 2 (green) included terms related to the methodology of OSCE evaluation: training, group, course, teaching, simulation, learning, difference, intervention, and confidence. Cluster 3 (red) included terms referring to student’s perception and experience: survey, interview, perception, anxiety, challenge, and preparation. The list of the different terms included in each cluster, and their corresponding statistical measurements, are detailed in the supplementary Table S1.

The VOSviewer software was used to perform a term co-occurrence map based on text data extracted from the abstract field of Scopus, using the full counting method. The thickness of the line between two co-occurring terms is associated with closer links. Co-occurring terms were clustered, and clusters were identified using different colors (red, blue, and green).

Terms from clusters 1 and 2 were close in the network with many interconnecting lines, while very few connections were observed between these two clusters and cluster 3. Notably, compared to other terms, ‘anxiety’ and ‘stress’ were more isolated within this network, without connections with student’s performance.

### International scientific network

**Figure 3.**
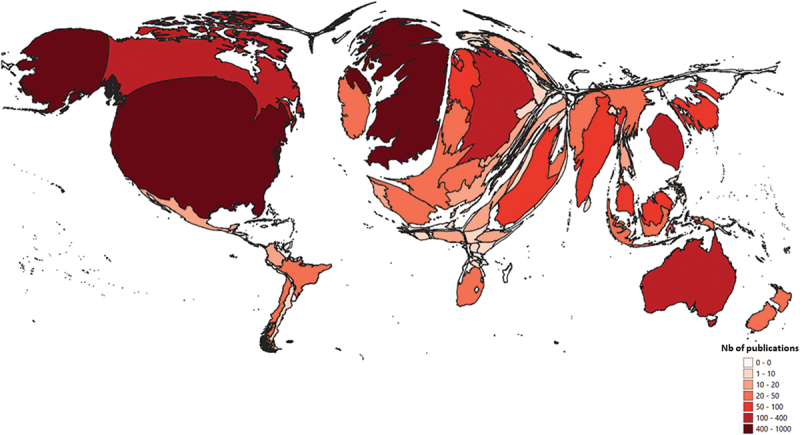
Density Equalizing Map Projection (DEMP) of countries contributing to OSCE research.

Among the 3,224 selected items, 3,117 originated from 108 different countries, and 107 (3.3%) could not be assigned to a specific country. Seven hundred and fourteen (22%) were from international collaborations involving a total of 89 different countries. The United States of America (USA), Canada, and the United Kingdom (UK) were the most productive countries, contributing in 923 (28.6%), 476 (14.8%), and 355 (11.0%) publications, respectively. The density equalizing mapping of this set of data also showed that the Netherlands, Germany, Saudi Arabia, Australia, and Taiwan significantly contributed to the research effort (Figure 3).

The DEMP was built using the total number of published items by each country.

**Figure 4.**
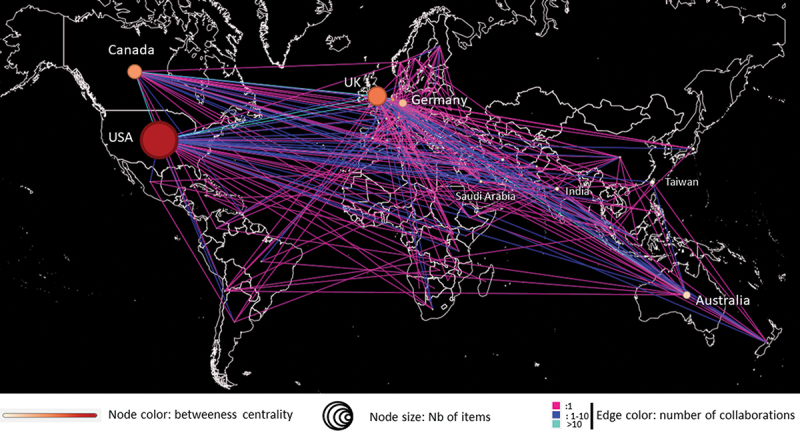
Network of international scientific collaboration in OSCE research.

International collaborative publications were analyzed in terms of scientific networks. The USA, UK, and Canada were the top three collaborating countries, in terms of number of items as well as betweenness centrality (Figure 4). In addition to an important interconnection between these three countries, with more than 10 collaborative studies, they were also individually connected to many other countries worldwide. Some discrepancies emerged between the top 10 contributor countries and the top 10 producer countries (Table 3). Some countries, such as Italy, Trinidad & Tobago and Belgium, were major contributors, but not among the most productive. In contrast, other countries, such as India, Taiwan, and Japan with a high number of published items, had few connections with other countries.

International scientific network was built using Table2Net and then exported to the Gephi software where the ‘Map of countries’ and ‘geolayout’ were used to visualize the network. The network includes nodes (countries) as well as edges (connection of two countries in a collaborative work). Node size and color are defined by the number of published items and betweenness centrality, respectively.

**Table 3.** Top 10 countries contributing to research in OSCE.

Rank	Country	Items (N)	Country	International collaborations (N)	Country	betweeness centrality
1	**united states**	923	**united states**	50	**united states**	1150.9
2	**united kingdom**	476	**united kingdom**	43	**united kingdom**	661.0
3	**canada**	355	**canada**	34	**canada**	516.7
4	**germany**	198	**netherlands**	34	**netherlands**	500.6
5	**australia**	184	**germany**	24	**germany**	298.9
6	taiwan	101	**australia**	24	trinidad and tobago	249.0
7	india	92	switzerland	22	belgium	209.5
8	**netherlands**	88	**saudi arabia**	20	italy	155.4
9	**saudi arabia**	74	belgium	19	**saudi arabia**	148.2
10	japan	72	italy	17	**australia**	145.3

Countries, ranked in the top 10 most prominent countries, regarding the total number of published items and its collaborative activities (number of collaborative studies and betweenness centrality), are in bold.

Finally, the analysis revealed that Asia was poorly connected within this international scientific network on research in OSCE.

## Discussion

To the best of our knowledge, this is the first bibliometric and scientometric analysis of educational research publications in the field of OSCE. This study confirms our assumption that OSCE is experiencing a recent paradigm shift towards its methodology and topics of research and its related scientific international network. Compared to previous study, our study provides novel insights and clues to promote research in OSCE, through different original observations. First, in the context of flourished debates regarding OSCE evolution, we noticed a recent increase in the number of highly cited articles focusing on remote OSCE. Moreover, we observed a lack of important connections between research related to student’s perception and research related to reliability, performance, and methodology of OSCE. Finally, besides the confirmation of a strong leadership from the USA, UK, and Canada in terms of volume of publications and collaborations, our study also highlighted an important scientific contribution of other countries which are scarcely connected within the international network. This point is even more important regarding the development of virtual/remote OSCE worldwide, to promote international guidelines on this new type of OSCE.

Although these results suggest several areas for improvement in OSCE research and organization, our study has some limitations. First, some countries, especially from Latin America and Africa, could also publish items in non-English language journals indexed in other databases than Scopus, such as SciELO (Scientific Electronic Library Online), that could underestimate the scientific contribution of those countries. Moreover, it is noteworthy that our study does not provide an in-depth look at the actual research being published as done in a systematic review, but rather records the locations and fields in which research relating to our topic is being published. However, we used quantitative parameters for co-occurrent terms and scientific networks, in order to limit subjectivity in data interpretation. It is through this broad analysis that the leading fields in this topic and the gaps between them become apparent, allowing us to propose what we believe to be worthwhile solutions.

The sudden increase in OSCE-related publications from 2019 occurred at the beginning of the COVID-19 pandemic (Figure 1). Between 2020 and 2024, 70% of the 10 most cited studies were related to virtual/remote OSCE organization, highlighting the growing and persistent interest of the scientific community for this alternative method. Although our study does not allow establishing a causal link between these two observations, it is plausible that the pandemic may have prompted the development of research in this field. However, the data comparing remote/virtual to live OSCE are scarce, and only one recent study reported no significant difference between these two OSCE modalities [32]. These observations call for additional studies assessing the feasibility and reliability of remote-OSCE. In addition, the development of consensual international guidelines could promote virtual and remote OSCE sharing between universities and countries.

The co-occurrence term network showed a gap between research on the modalities of OSCE and the student’s perception suggested a need for more studies on the association between performance/reliability of OSCE and student’s perception/experience or mental health (e.g., anxiety). Although co-occurrence term networks could appeal to subjective data interpretation at first glance, this network is based on a computational method, ranking terms according to the number of occurrences. In order to provide a more quantitative and objective analysis of our results, we selected the top 15 most co-occurrent terms for each cluster. Thus, anxiety was identified as one of the most co-occurrent terms for the cluster 3. Together with other consistent terms from this cluster, such as ‘stress’, ‘perception’, ‘student’s perception’, and ‘fairness’, we emphasized the importance of student’s perception in this research cluster. The lack of connections between cluster 3 and the clusters 1 and 2 suggests the lack of studies linking student’s perception (e.g., anxiety) to OSCE performance. This observation is important when considering that medical students represent a population at high-risk of mental disorders including anxiety and depression [33–35]. In France, 20.5% are regular anxiolytic consumers and 12.5% are followed-up by a psychiatrist or psychologist [36]. OSCE involves the use of body and voice, directly impacting physiological parameters such as heart rate respiratory variation, which is related to anxiety. Besides, the live interaction with a standardized patient during OSCE might increase the amplitude of emotional reactions and contribute to generating greater anxiety than written exams. The level of anxiety is high in undergraduate medical students during OSCE [37], but the effects of anxiety on students’ performance are still debated [37–39]. In our institution, we organize monthly open workshops to help under-graduate medical students in mitigating their level of anxiety. Our results emphasize this research gap between anxiety and OSCE performance and suggest that future investigations should address this issue. Describing the relationship between anxiety and performance during OSCE might allow improving students’ preparation and training.

Finally, the international scientific network appeared to be led by the United States of America, the United Kingdom, and Canada and to have expanded mainly to European countries, Saudi Arabia, and Australia. Although Taiwan, India, and Japan are important individual contributors to OSCE publications, international collaborative studies involving Asian countries are lacking. Based on the results of betweenness centrality analysis, the United States, United Kingdom, Canada, and the Netherlands were identified as the most influential countries that could improve the future development of the international research network in OSCE.

In conclusion, this overall landscape of OSCE publications identified several clues to promote OSCE educational research. Studies comparing live and remote OSCE are needed to complete the assessment of this recently developed method. International recommendations on remote OSCE would promote collaboration and sharing between universities across the world. Although the interplay between stress and performance is well demonstrated in other educational contexts, our study suggests the need for additional studies investigating the relationship between anxiety and performance during OSCE. With the support of leading countries in this field, the international research network on OSCE could be developed to promote larger collaborative research projects.

## Supplementary Material

Supplementary Table 1.docx
