# Supplementary material for: Clues for improvement of research in objective structured clinical examination
Source: Med Educ Online. 2024 Jun 27;29(1):2370617. doi: 10.1080/10872981.2024.2370617 (PMC11212575; doi:10.1080/10872981.2024.2370617)
Supplement: Supplementary Table 1.docx [file ZMEO_A_2370617_SM5410.docx]

**Supplementary Table 1. List of terms included in each cluster within the co-occurrence term network (figure 2)**

| Term | cluster | weight<Links> | weight<Total link strength> | weight<Occurrences> | score<Avg. pub. year> | score<Avg. citations> | score<Avg. norm. citations> |
| --- | --- | --- | --- | --- | --- | --- | --- |
| station | 1 | 325 | 32797 | 1695 | 2011.3 | 20.451 | 0.9417 |
| resident | 1 | 308 | 30524 | 1437 | 2012.7 | 14.913 | 0.8792 |
| examiner | 1 | 318 | 17397 | 867 | 2014.5 | 15.156 | 1.182 |
| reliability | 1 | 320 | 17223 | 845 | 2010.6 | 31.291 | 1.2234 |
| communication skill | 1 | 319 | 17076 | 749 | 2014.6 | 21 | 1.1987 |
| validity | 1 | 314 | 13134 | 647 | 2011.9 | 28.345 | 1.2856 |
| checklist | 1 | 317 | 12933 | 572 | 2013.4 | 16.224 | 0.991 |
| communication | 1 | 320 | 12636 | 565 | 2015.4 | 15.935 | 1.0096 |
| correlation | 1 | 318 | 12007 | 558 | 2012.7 | 19.715 | 0.9741 |
| item | 1 | 319 | 11313 | 508 | 2013.6 | 14.081 | 0.8433 |
| osce score | 1 | 316 | 10481 | 466 | 2014.2 | 15.472 | 0.9419 |
| clerkship | 1 | 294 | 9159 | 457 | 2011.5 | 15.93 | 0.8579 |
| rating | 1 | 309 | 9837 | 447 | 2011.4 | 25.588 | 1.1142 |
| evidence | 1 | 322 | 9216 | 421 | 2014 | 20.437 | 1.2874 |
| standardized patient | 1 | 312 | 8464 | 421 | 2011.9 | 23.159 | 1.0349 |
| clinical competence | 1 | 315 | 7550 | 412 | 2010.7 | 28.655 | 1.1631 |
| candidate | 1 | 297 | 7902 | 409 | 2011.6 | 17.734 | 1.4283 |
| trainee | 1 | 309 | 8324 | 397 | 2015.2 | 10.914 | 0.77 |
| grade | 1 | 301 | 7105 | 332 | 2014.4 | 13.124 | 0.919 |
| relationship | 1 | 306 | 6629 | 320 | 2014.1 | 18.166 | 1.041 |
| osce station | 1 | 318 | 6664 | 316 | 2013 | 17.835 | 0.9136 |
| standard | 1 | 307 | 5507 | 299 | 2011.7 | 22.926 | 1.0131 |
| mark | 1 | 282 | 5788 | 287 | 2009.9 | 14.206 | 0.6769 |
| encounter | 1 | 295 | 6133 | 273 | 2014.3 | 14.63 | 0.8702 |
| student performance | 1 | 308 | 5344 | 273 | 2012.1 | 20.048 | 1.0039 |
| assessment tool | 1 | 301 | 5160 | 265 | 2015 | 17.63 | 0.9471 |
| rater | 1 | 269 | 5427 | 253 | 2013.4 | 21.526 | 0.8715 |
| instrument | 1 | 301 | 5365 | 251 | 2012.3 | 23.056 | 1.0901 |
| decision | 1 | 295 | 4457 | 216 | 2015 | 21.375 | 1.1991 |
| physical examination | 1 | 286 | 4725 | 212 | 2011.9 | 16.943 | 0.9352 |
| expert | 1 | 289 | 4658 | 200 | 2014.8 | 16.91 | 0.9788 |
| criterium | 1 | 297 | 4243 | 196 | 2014.4 | 16.158 | 1.2012 |
| variance | 1 | 289 | 4201 | 186 | 2012.2 | 21.43 | 1.1028 |
| interaction | 1 | 300 | 4053 | 178 | 2014.3 | 23.14 | 1.3697 |
| empathy | 1 | 230 | 3706 | 167 | 2015.2 | 35.228 | 2.142 |
| gender | 1 | 273 | 3549 | 167 | 2015.5 | 15.814 | 1.0964 |
| agreement | 1 | 267 | 3961 | 166 | 2014.1 | 16.566 | 0.931 |
| fellow | 1 | 193 | 3252 | 166 | 2016.2 | 8.3253 | 0.5525 |
| assessor | 1 | 263 | 3762 | 162 | 2016.9 | 12.796 | 1.0556 |
| behavior | 1 | 267 | 3393 | 161 | 2013.8 | 18.267 | 0.9255 |
| osce performance | 1 | 287 | 3603 | 161 | 2015.7 | 15.727 | 1.099 |
| error | 1 | 257 | 2813 | 143 | 2011.4 | 30.035 | 1.2112 |
| accuracy | 1 | 249 | 2649 | 142 | 2015.2 | 21.845 | 1.0831 |
| cronbach | 1 | 284 | 3300 | 139 | 2014.5 | 12.237 | 0.7159 |
| professionalism | 1 | 257 | 3009 | 136 | 2014.4 | 19.588 | 1.0737 |
| measurement | 1 | 280 | 2725 | 131 | 2010.9 | 34.153 | 1.3983 |
| source | 1 | 273 | 3014 | 129 | 2014.6 | 33.605 | 1.4681 |
| examinee | 1 | 255 | 2747 | 128 | 2013.4 | 18.32 | 0.7986 |
| sps | 1 | 276 | 2669 | 124 | 2013.3 | 25.919 | 1.1452 |
| total score | 1 | 275 | 2901 | 124 | 2015.4 | 14.411 | 1.1525 |
| average | 1 | 286 | 2670 | 123 | 2014 | 16.984 | 0.9306 |
| pgy | 1 | 185 | 2851 | 121 | 2014.5 | 13.025 | 1.4545 |
| comment | 1 | 241 | 2383 | 115 | 2014.3 | 11.183 | 0.8523 |
| academic year | 1 | 262 | 2554 | 113 | 2014 | 14.673 | 0.8857 |
| decision making | 1 | 265 | 2310 | 112 | 2016.4 | 17.714 | 1.0393 |
| observer | 1 | 243 | 2542 | 111 | 2010.7 | 19.387 | 0.8524 |
| p0001 | 1 | 237 | 2699 | 107 | 2014.9 | 12.084 | 0.7275 |
| pass | 1 | 234 | 2380 | 107 | 2013.6 | 17.29 | 0.918 |
| rating scale | 1 | 227 | 2665 | 106 | 2015.3 | 24.604 | 1.7029 |
| risk | 1 | 250 | 2047 | 106 | 2015.7 | 19.104 | 1.2175 |
| canada | 1 | 234 | 1905 | 105 | 2008.9 | 18.867 | 0.7796 |
| influence | 1 | 258 | 2088 | 105 | 2014.2 | 12.514 | 0.8656 |
| residency | 1 | 234 | 2297 | 103 | 2012.4 | 19.32 | 0.9471 |
| rubric | 1 | 234 | 1926 | 103 | 2019 | 11.214 | 0.8688 |
| inter rater reliability | 1 | 228 | 2282 | 101 | 2014.7 | 16.644 | 1.0404 |
| alpha | 1 | 260 | 2430 | 100 | 2015 | 18.19 | 1.0626 |
| variation | 1 | 266 | 2241 | 100 | 2012.7 | 29.73 | 1.2107 |
| third year medical student | 1 | 236 | 2059 | 99 | 2011.2 | 13.475 | 0.8451 |
| academic performance | 1 | 208 | 1883 | 98 | 2017 | 12.571 | 0.9053 |
| strength | 1 | 273 | 1932 | 96 | 2014.5 | 17.594 | 1.064 |
| clinical experience | 1 | 230 | 2133 | 95 | 2010.9 | 21.874 | 1.0863 |
| achievement | 1 | 243 | 1944 | 94 | 2014.7 | 17.638 | 1.2653 |
| evaluator | 1 | 201 | 2200 | 92 | 2011.4 | 9.9783 | 0.5515 |
| actor | 1 | 227 | 1827 | 91 | 2015.5 | 12.198 | 1.6674 |
| core competency | 1 | 226 | 2132 | 91 | 2013.5 | 21.528 | 1.0979 |
| global rating | 1 | 200 | 2161 | 91 | 2008.1 | 45.275 | 1.4089 |
| internal consistency | 1 | 231 | 2075 | 89 | 2015.2 | 13.348 | 0.7456 |
| internal medicine | 1 | 249 | 1956 | 88 | 2012.5 | 17.386 | 0.9995 |
| program director | 1 | 240 | 2115 | 87 | 2014.4 | 13.218 | 0.7614 |
| simulated patient | 1 | 244 | 1617 | 87 | 2014.3 | 25.494 | 1.0297 |
| acceptability | 1 | 247 | 1846 | 86 | 2016.2 | 13.733 | 0.999 |
| station osce | 1 | 261 | 1762 | 85 | 2010.8 | 25.612 | 1.2536 |
| weakness | 1 | 255 | 1798 | 84 | 2013.3 | 17.155 | 0.9423 |
| selection | 1 | 223 | 1521 | 83 | 2013.7 | 24.578 | 1.2992 |
| clinical evaluation | 1 | 217 | 1380 | 80 | 2011.2 | 15.4 | 0.7391 |
| interrater reliability | 1 | 207 | 1820 | 80 | 2012.2 | 18.938 | 0.8551 |
| female | 1 | 237 | 1842 | 79 | 2015.5 | 13.734 | 0.9132 |
| male | 1 | 232 | 1811 | 79 | 2014.5 | 14.177 | 0.8335 |
| average score | 1 | 228 | 1742 | 75 | 2015.9 | 10.387 | 1.1804 |
| validity evidence | 1 | 187 | 1530 | 74 | 2015.9 | 27.554 | 1.5017 |
| significant correlation | 1 | 226 | 1505 | 73 | 2011.5 | 15.616 | 0.7829 |
| consistency | 1 | 218 | 1736 | 72 | 2013.7 | 43.681 | 1.2992 |
| mmi | 1 | 115 | 1697 | 72 | 2014.3 | 58.361 | 2.2297 |
| objectivity | 1 | 215 | 1389 | 72 | 2014.9 | 18.694 | 1.0669 |
| validation | 1 | 238 | 1616 | 72 | 2016.2 | 11.389 | 0.7896 |
| failure | 1 | 218 | 1368 | 69 | 2014.8 | 12.884 | 0.7394 |
| postgraduate year | 1 | 198 | 1823 | 68 | 2014.4 | 16.574 | 0.9716 |
| global rating scale | 1 | 185 | 1561 | 67 | 2015.5 | 16.597 | 1.0279 |
| surgical resident | 1 | 150 | 1467 | 67 | 2006.1 | 34.508 | 1.1314 |
| interpersonal skill | 1 | 196 | 1479 | 66 | 2009.2 | 40.894 | 1.4664 |
| preceptor | 1 | 167 | 1363 | 66 | 2010.3 | 9.3636 | 0.6367 |
| real patient | 1 | 197 | 1257 | 66 | 2013.4 | 17.212 | 0.8698 |
| evaluation method | 1 | 207 | 1229 | 65 | 2009.9 | 16.462 | 0.8524 |
| extent | 1 | 236 | 1295 | 65 | 2013.9 | 20.754 | 1.1891 |
| long case | 1 | 130 | 1170 | 64 | 2007.4 | 33.094 | 1.0457 |
| transition | 1 | 216 | 1354 | 64 | 2016 | 18.172 | 1.5679 |
| content validity | 1 | 190 | 1361 | 63 | 2012.1 | 21.73 | 1.0146 |
| general practice | 1 | 180 | 1189 | 63 | 2008.7 | 21.254 | 1.245 |
| third year | 1 | 214 | 1330 | 61 | 2012.3 | 36.377 | 1.3086 |
| station objective structured clinical examination | 1 | 240 | 1275 | 60 | 2010.5 | 26.1 | 1.1538 |
| taylor & francis group | 1 | 238 | 1174 | 60 | 2019 | 11.667 | 1.9063 |
| graduate medical education | 1 | 214 | 1444 | 59 | 2013.1 | 24.22 | 1.195 |
| national board | 1 | 190 | 1095 | 58 | 2007.9 | 22.172 | 0.9862 |
| predictive validity | 1 | 149 | 1146 | 58 | 2014.9 | 25.431 | 1.383 |
| formative osce | 1 | 170 | 1395 | 56 | 2017.7 | 11.714 | 0.9906 |
| overall score | 1 | 225 | 1258 | 56 | 2012.8 | 23.625 | 1.2518 |
| psychometric property | 1 | 185 | 1053 | 56 | 2013.7 | 21.071 | 1.2394 |
| accreditation council | 1 | 206 | 1300 | 55 | 2013.3 | 21.455 | 1.0386 |
| osce result | 1 | 192 | 1202 | 54 | 2014.1 | 12.13 | 0.8672 |
| blackwell publishing ltd | 1 | 215 | 1196 | 53 | 2008.9 | 42.377 | 1.7601 |
| entry | 1 | 202 | 1088 | 53 | 2012.5 | 26.019 | 1.1836 |
| video recording | 1 | 180 | 1233 | 52 | 2017.2 | 10.673 | 0.9065 |
| second year | 1 | 195 | 1083 | 50 | 2009.8 | 17.34 | 0.9557 |
| standard setting | 1 | 161 | 791 | 50 | 2011.5 | 28.92 | 1.0154 |
| summative osce | 1 | 188 | 936 | 50 | 2016.1 | 16.38 | 1.2734 |
| american board | 1 | 166 | 961 | 48 | 2010.6 | 20.958 | 1.0543 |
| standardised patient | 1 | 145 | 949 | 47 | 2010.7 | 42.468 | 1.3779 |
| immediate feedback | 1 | 188 | 926 | 45 | 2009.2 | 29.689 | 1.3622 |
| informed consent | 1 | 186 | 1009 | 44 | 2012.5 | 13.114 | 0.6559 |
| differential diagnosis | 1 | 167 | 709 | 42 | 2014.7 | 8.0238 | 0.6839 |
| informa uk limited | 1 | 202 | 774 | 42 | 2019.7 | 12.762 | 2.2627 |
| senior resident | 1 | 133 | 1136 | 42 | 2010 | 26.357 | 1.153 |
| royal college | 1 | 164 | 726 | 41 | 2013.4 | 5.878 | 1.0686 |
| generalizability theory | 1 | 142 | 895 | 40 | 2010.4 | 43.4 | 1.384 |
| training | 2 | 325 | 45528 | 2153 | 2015.5 | 17.059 | 1.216 |
| group | 2 | 324 | 39537 | 1731 | 2014.3 | 16.444 | 1.1233 |
| knowledge | 2 | 325 | 26891 | 1237 | 2014.7 | 15.437 | 1.0184 |
| course | 2 | 322 | 22510 | 1111 | 2013.8 | 13.957 | 0.9648 |
| curriculum | 2 | 322 | 20276 | 958 | 2013.8 | 16.327 | 1.041 |
| teaching | 2 | 325 | 18245 | 831 | 2014.2 | 17.338 | 1.1607 |
| simulation | 2 | 316 | 14400 | 655 | 2016.8 | 17.643 | 1.3137 |
| learning | 2 | 323 | 15095 | 650 | 2015.7 | 15.402 | 1.3847 |
| difference | 2 | 324 | 14598 | 624 | 2014 | 22.941 | 1.2394 |
| care | 2 | 312 | 11863 | 533 | 2015.2 | 12.041 | 0.8942 |
| session | 2 | 318 | 11092 | 466 | 2014.9 | 13.41 | 1.064 |
| intervention | 2 | 317 | 11197 | 452 | 2015.8 | 15.215 | 1.0083 |
| confidence | 2 | 312 | 10802 | 451 | 2016.7 | 18.051 | 1.4502 |
| physician | 2 | 307 | 9595 | 451 | 2011.3 | 17.018 | 0.8949 |
| effectiveness | 2 | 316 | 10091 | 422 | 2015.3 | 16.787 | 1.1336 |
| point | 2 | 319 | 10087 | 399 | 2016.3 | 14.216 | 1.0231 |
| self | 2 | 309 | 9290 | 382 | 2016 | 16.411 | 1.0981 |
| significant difference | 2 | 323 | 8904 | 377 | 2014.7 | 14.324 | 0.9295 |
| video | 2 | 296 | 8782 | 376 | 2018.2 | 12.513 | 1.2481 |
| control group | 2 | 292 | 9392 | 363 | 2015.8 | 14.964 | 1.0563 |
| nurse | 2 | 275 | 7718 | 353 | 2016.6 | 14.637 | 1.0402 |
| module | 2 | 295 | 7866 | 333 | 2015.3 | 10.964 | 0.9049 |
| hospital | 2 | 306 | 6881 | 298 | 2013.4 | 17.617 | 1.0371 |
| month | 2 | 305 | 7300 | 282 | 2014.8 | 19.383 | 1.3869 |
| workshop | 2 | 280 | 5942 | 277 | 2014.4 | 10.065 | 0.729 |
| rotation | 2 | 288 | 6194 | 258 | 2012.7 | 14.419 | 0.8625 |
| resource | 2 | 309 | 5307 | 254 | 2015.3 | 14.866 | 0.9659 |
| nursing student | 2 | 249 | 4856 | 244 | 2017.9 | 15.541 | 1.5343 |
| lecture | 2 | 287 | 5846 | 236 | 2016.5 | 15.814 | 1.2324 |
| class | 2 | 293 | 4664 | 233 | 2013.1 | 14.107 | 1.1741 |
| intern | 2 | 236 | 5360 | 232 | 2013.1 | 14.655 | 0.71 |
| training program | 2 | 284 | 5097 | 224 | 2015.1 | 12.768 | 0.8692 |
| trial | 2 | 286 | 5537 | 222 | 2015.5 | 22.883 | 1.5163 |
| peer | 2 | 297 | 5523 | 221 | 2016.1 | 19.919 | 1.4299 |
| pre | 2 | 295 | 5638 | 217 | 2016 | 11.959 | 0.9637 |
| instruction | 2 | 291 | 4215 | 197 | 2012.5 | 22.523 | 1.3377 |
| teacher | 2 | 284 | 4317 | 197 | 2014 | 14.624 | 0.9018 |
| acquisition | 2 | 288 | 4359 | 195 | 2013.8 | 16.492 | 1.1046 |
| intervention group | 2 | 258 | 4947 | 190 | 2016.9 | 13.221 | 1.0004 |
| week | 2 | 288 | 4453 | 180 | 2015 | 11.3 | 0.8554 |
| simulator | 2 | 248 | 3507 | 175 | 2014.4 | 16.914 | 0.9941 |
| control | 2 | 272 | 4324 | 163 | 2015 | 19.301 | 1.2149 |
| hand | 2 | 255 | 3679 | 158 | 2015.8 | 11.968 | 0.8547 |
| technology | 2 | 269 | 2930 | 157 | 2016.9 | 18.223 | 1.4038 |
| tutor | 2 | 240 | 3422 | 141 | 2013.5 | 17.979 | 1.1853 |
| mcq | 2 | 260 | 3112 | 138 | 2011.1 | 15.58 | 0.9025 |
| semester | 2 | 269 | 2927 | 136 | 2016.6 | 9 | 0.8244 |
| ultrasound | 2 | 197 | 3377 | 136 | 2018.8 | 15.177 | 1.6185 |
| instructor | 2 | 237 | 3148 | 134 | 2015.9 | 11.179 | 1.1004 |
| multiple choice question | 2 | 283 | 3024 | 134 | 2012.7 | 21.179 | 1.227 |
| t test | 2 | 278 | 3222 | 134 | 2015.8 | 14.605 | 1.0503 |
| age | 2 | 277 | 3157 | 133 | 2016.2 | 13.233 | 0.9833 |
| provider | 2 | 231 | 3345 | 132 | 2018.5 | 11.78 | 0.9642 |
| practical skill | 2 | 258 | 2672 | 127 | 2016.7 | 8.6693 | 0.771 |
| web | 2 | 234 | 3007 | 123 | 2014.7 | 21.772 | 1.2768 |
| exposure | 2 | 271 | 2911 | 122 | 2016.2 | 9.3115 | 0.8648 |
| interest | 2 | 276 | 3008 | 117 | 2014.3 | 24.034 | 1.1537 |
| reflection | 2 | 244 | 2514 | 117 | 2015.6 | 20.154 | 1.1087 |
| obstetric | 2 | 237 | 2874 | 114 | 2011.1 | 11.667 | 1.164 |
| increase | 2 | 282 | 2470 | 110 | 2013.6 | 16.891 | 1.0917 |
| treatment | 2 | 267 | 2116 | 109 | 2013.7 | 17.762 | 1.3739 |
| self efficacy | 2 | 161 | 2424 | 101 | 2016.1 | 20.386 | 1.313 |
| significant improvement | 2 | 250 | 2453 | 101 | 2014.2 | 15.762 | 1.0249 |
| undergraduate medical student | 2 | 251 | 2167 | 98 | 2016.2 | 11.725 | 0.9517 |
| internship | 2 | 216 | 2045 | 97 | 2014.7 | 8.7629 | 0.6431 |
| efficacy | 2 | 249 | 2153 | 94 | 2015.3 | 13.319 | 1.3527 |
| experimental group | 2 | 176 | 2295 | 89 | 2017 | 29.449 | 1.7608 |
| access | 2 | 241 | 1911 | 75 | 2017.3 | 19.947 | 1.2586 |
| midwife | 2 | 172 | 1693 | 74 | 2017.2 | 20.46 | 1.4725 |
| practical examination | 2 | 207 | 1498 | 74 | 2013.2 | 9.6081 | 0.6278 |
| procedural skill | 2 | 227 | 1762 | 74 | 2015.6 | 15.257 | 1.0341 |
| post test | 2 | 205 | 1940 | 72 | 2016.2 | 11.014 | 0.9244 |
| life | 2 | 240 | 1625 | 71 | 2016.2 | 14.747 | 1.0456 |
| undergraduate student | 2 | 227 | 1361 | 71 | 2016 | 12.423 | 0.8268 |
| fourth year medical student | 2 | 220 | 1538 | 70 | 2013.5 | 16.014 | 1.1998 |
| india | 2 | 189 | 1640 | 70 | 2016.6 | 10.671 | 0.8165 |
| bad news | 2 | 170 | 1677 | 69 | 2014.5 | 21.971 | 1.209 |
| woman | 2 | 229 | 1615 | 69 | 2014.4 | 10.725 | 0.853 |
| technical skill | 2 | 223 | 1545 | 68 | 2013.9 | 22.721 | 1.2906 |
| midwifery student | 2 | 145 | 1355 | 65 | 2016.8 | 13.246 | 0.9608 |
| pbl | 2 | 157 | 1196 | 65 | 2011.3 | 12.262 | 0.822 |
| taiwan | 2 | 189 | 1588 | 65 | 2016.9 | 8.4462 | 0.8769 |
| germany | 2 | 198 | 1383 | 64 | 2014.2 | 10.047 | 0.7153 |
| educational intervention | 2 | 205 | 1361 | 63 | 2015.2 | 13.159 | 0.8773 |
| iqr | 2 | 159 | 1687 | 63 | 2020.1 | 5.9683 | 1.1943 |
| patient safety | 2 | 206 | 1382 | 60 | 2015.5 | 13.267 | 0.7744 |
| baseline | 2 | 176 | 1631 | 59 | 2016.3 | 16.407 | 1.1101 |
| higher score | 2 | 210 | 1214 | 57 | 2014.8 | 11.368 | 0.8627 |
| emergency medicine | 2 | 191 | 1348 | 56 | 2011.4 | 20.036 | 1.1272 |
| gynecology | 2 | 199 | 1458 | 56 | 2015 | 8.75 | 1.1388 |
| median | 2 | 197 | 1603 | 56 | 2018.1 | 10.286 | 1.3915 |
| p value | 2 | 220 | 1478 | 55 | 2018.6 | 5.8909 | 0.695 |
| medical faculty | 2 | 182 | 1158 | 54 | 2014.3 | 15.204 | 1.1323 |
| motivation | 2 | 209 | 1279 | 54 | 2014.5 | 8.0185 | 1.0501 |
| experimental study | 2 | 199 | 1385 | 53 | 2018.9 | 15.038 | 1.7302 |
| e learning | 2 | 158 | 1243 | 52 | 2019.4 | 10.039 | 1.5902 |
| mcqs | 2 | 163 | 1214 | 52 | 2014.5 | 11.442 | 0.9063 |
| pretest | 2 | 183 | 1304 | 52 | 2015 | 13.327 | 1.0119 |
| theoretical knowledge | 2 | 186 | 1005 | 52 | 2015.9 | 10.5 | 0.8464 |
| beginning | 2 | 218 | 1315 | 50 | 2014.5 | 28.38 | 1.2338 |
| trauma | 2 | 170 | 1213 | 49 | 2010.4 | 17.082 | 1.0589 |
| general practitioner | 2 | 159 | 825 | 48 | 2010 | 11.854 | 0.5114 |
| july | 2 | 212 | 1216 | 48 | 2017.6 | 8.5625 | 0.8311 |
| pal | 2 | 103 | 1226 | 48 | 2015.4 | 33.25 | 1.8331 |
| small group | 2 | 211 | 1064 | 48 | 2009.3 | 27.479 | 1.072 |
| clinical simulation | 2 | 136 | 803 | 47 | 2017.2 | 19.553 | 1.6226 |
| study group | 2 | 165 | 1243 | 47 | 2011.6 | 17.766 | 1.0024 |
| significant increase | 2 | 208 | 1096 | 46 | 2014.9 | 14.022 | 1.179 |
| p001 | 2 | 196 | 1165 | 44 | 2015 | 16.568 | 1.0205 |
| process | 3 | 322 | 11700 | 612 | 2013.8 | 17.427 | 1.1185 |
| survey | 3 | 323 | 11468 | 516 | 2016.8 | 12.979 | 1.3169 |
| interview | 3 | 307 | 7283 | 373 | 2014.9 | 26.182 | 1.2135 |
| perception | 3 | 311 | 7800 | 365 | 2016.4 | 13.167 | 1.0544 |
| article | 3 | 309 | 6647 | 341 | 2015.5 | 21.557 | 1.5884 |
| review | 3 | 308 | 6283 | 304 | 2016 | 25.895 | 1.7392 |
| paper | 3 | 303 | 5215 | 300 | 2011 | 26.267 | 1.2772 |
| assessment method | 3 | 303 | 5136 | 264 | 2015 | 16.046 | 1.2972 |
| activity | 3 | 311 | 5476 | 262 | 2016.3 | 12.687 | 0.9495 |
| covid | 3 | 283 | 5269 | 260 | 2021.9 | 8.6923 | 2.0284 |
| anxiety | 3 | 256 | 4306 | 235 | 2017.5 | 16.885 | 1.506 |
| challenge | 3 | 308 | 4198 | 219 | 2016.4 | 18.671 | 1.3287 |
| preparation | 3 | 286 | 4331 | 219 | 2015.5 | 14.749 | 1.0843 |
| literature | 3 | 302 | 4482 | 208 | 2013.5 | 33.183 | 1.6565 |
| framework | 3 | 296 | 4018 | 204 | 2018 | 13.392 | 1.0135 |
| perspective | 3 | 288 | 4229 | 203 | 2016.6 | 13.808 | 1.1112 |
| curricula | 3 | 301 | 4200 | 198 | 2014 | 23.793 | 1.3035 |
| pandemic | 3 | 270 | 4004 | 193 | 2021.9 | 8.4611 | 2.1044 |
| theme | 3 | 283 | 4077 | 183 | 2017.7 | 9.8907 | 1.1678 |
| clinical practice | 3 | 294 | 3576 | 170 | 2016.7 | 13.965 | 1.0217 |
| pharmacist | 3 | 222 | 3285 | 169 | 2015.5 | 8.5976 | 0.5952 |
| nursing | 3 | 259 | 3240 | 167 | 2014.9 | 22.126 | 1.3915 |
| percent | 3 | 246 | 3302 | 165 | 2012 | 20.321 | 0.9747 |
| person | 3 | 285 | 3544 | 164 | 2018.1 | 12.793 | 1.3651 |
| face | 3 | 260 | 3483 | 163 | 2019.3 | 8.9693 | 1.4805 |
| health | 3 | 278 | 3412 | 157 | 2016.6 | 12.733 | 0.9969 |
| delivery | 3 | 281 | 3419 | 155 | 2017.2 | 10.084 | 1.1361 |
| project | 3 | 281 | 3026 | 147 | 2014.3 | 12.85 | 0.697 |
| topic | 3 | 277 | 2884 | 144 | 2015.6 | 9.7361 | 0.8266 |
| country | 3 | 281 | 3227 | 143 | 2017.5 | 9.986 | 1.1582 |
| pharmacy student | 3 | 230 | 2477 | 134 | 2018.4 | 8.6642 | 0.99 |
| view | 3 | 273 | 2525 | 131 | 2015.8 | 11.298 | 1.1058 |
| advantage | 3 | 273 | 2528 | 125 | 2012.4 | 21.36 | 1.3836 |
| opinion | 3 | 269 | 2504 | 117 | 2012.6 | 15.068 | 0.7528 |
| clinical assessment | 3 | 252 | 2195 | 114 | 2015 | 13.956 | 1.1301 |
| pharmacy | 3 | 227 | 2036 | 113 | 2015.7 | 11.089 | 0.7492 |
| psychiatry | 3 | 212 | 1764 | 112 | 2011 | 15.509 | 0.9788 |
| integration | 3 | 262 | 2271 | 110 | 2014.5 | 10.936 | 0.9681 |
| participation | 3 | 264 | 2628 | 109 | 2014 | 14.523 | 0.9353 |
| stress | 3 | 247 | 2187 | 108 | 2018.8 | 10.62 | 1.1406 |
| formative assessment | 3 | 257 | 2282 | 107 | 2013.9 | 10.15 | 0.7299 |
| dental student | 3 | 218 | 1931 | 104 | 2015.8 | 12.087 | 0.9468 |
| dentistry | 3 | 213 | 2022 | 104 | 2013.7 | 13.923 | 0.9014 |
| students perception | 3 | 239 | 2143 | 103 | 2016.6 | 15.233 | 1.2485 |
| discipline | 3 | 264 | 2038 | 103 | 2012.4 | 18.359 | 1.1302 |
| community | 3 | 241 | 2017 | 101 | 2011.2 | 12.238 | 0.7824 |
| focus group | 3 | 245 | 2125 | 100 | 2016.3 | 11.8 | 1.0818 |
| focus | 3 | 276 | 1894 | 99 | 2014.9 | 16.657 | 1.0434 |
| recommendation | 3 | 246 | 1755 | 93 | 2015.3 | 18.817 | 0.9604 |
| final year medical student | 3 | 227 | 1747 | 91 | 2012.5 | 24.857 | 1.6993 |
| barrier | 3 | 223 | 1746 | 89 | 2017.1 | 13.303 | 1.2759 |
| patient care | 3 | 261 | 1963 | 89 | 2014.8 | 13.944 | 1.0137 |
| summative assessment | 3 | 245 | 1785 | 87 | 2013.4 | 17.977 | 1.0994 |
| acceptance | 3 | 228 | 1722 | 84 | 2014.2 | 17.191 | 0.9777 |
| respondent | 3 | 233 | 1917 | 82 | 2015.8 | 16.207 | 1.7196 |
| expectation | 3 | 254 | 1853 | 82 | 2011.8 | 19.622 | 1.086 |
| example | 3 | 253 | 1524 | 80 | 2013.2 | 27.4 | 1.4529 |
| oral examination | 3 | 217 | 1727 | 80 | 2007.8 | 23.65 | 0.9886 |
| readiness | 3 | 225 | 1517 | 79 | 2018.2 | 9.8861 | 0.9359 |
| response rate | 3 | 251 | 1688 | 77 | 2014.8 | 11.935 | 0.8578 |
| undergraduate medical education | 3 | 217 | 1514 | 76 | 2014.8 | 16.632 | 1.314 |
| cross sectional study | 3 | 236 | 1617 | 76 | 2018.1 | 6.6447 | 0.6414 |
| insight | 3 | 242 | 1465 | 73 | 2015.7 | 11.397 | 0.95 |
| innovation | 3 | 225 | 1259 | 71 | 2012.4 | 16.268 | 0.9985 |
| consideration | 3 | 244 | 1402 | 69 | 2013.4 | 23.652 | 1.5407 |
| collaboration | 3 | 253 | 1376 | 69 | 2014.6 | 14.116 | 0.9198 |
| student perception | 3 | 227 | 1410 | 65 | 2016.2 | 15.108 | 0.9625 |
| vosce | 3 | 109 | 1157 | 61 | 2020.1 | 14.197 | 2.9358 |
| systematic review | 3 | 184 | 1433 | 60 | 2019.7 | 22.05 | 2.2024 |
| semi | 3 | 215 | 1334 | 60 | 2017.6 | 11.217 | 1.1483 |
| australia | 3 | 203 | 1074 | 57 | 2015.6 | 12.14 | 1.2096 |
| provision | 3 | 221 | 1392 | 57 | 2016.5 | 10.86 | 0.9315 |
| modification | 3 | 203 | 1143 | 56 | 2014.3 | 10.643 | 0.7546 |
| clinical placement | 3 | 169 | 868 | 55 | 2015.4 | 12.418 | 0.8364 |
| virtual osce | 3 | 153 | 985 | 51 | 2022.2 | 6.3137 | 2.0871 |
| disability | 3 | 83 | 1046 | 51 | 2018.6 | 12.98 | 1.3821 |
| workplace | 3 | 190 | 885 | 49 | 2018 | 11.204 | 1.3239 |
| pharmacy education | 3 | 149 | 909 | 48 | 2018.9 | 9.1875 | 0.8815 |
| medical educator | 3 | 216 | 975 | 46 | 2011.6 | 29.457 | 1.4853 |
| fairness | 3 | 179 | 908 | 43 | 2016.8 | 11.512 | 1.1508 |
| japan | 3 | 155 | 721 | 43 | 2014.7 | 14.698 | 0.6538 |
| final year | 3 | 192 | 764 | 42 | 2014.4 | 24.452 | 1.8873 |
| january | 3 | 220 | 941 | 40 | 2015.7 | 8.325 | 0.7584 |
